# Development of a Clinically Applicable NanoString-Based Gene Expression Classifier for Muscle-Invasive Bladder Cancer Molecular Stratification

**DOI:** 10.3390/cancers14194911

**Published:** 2022-10-07

**Authors:** Ekaterina Olkhov-Mitsel, Yanhong Yu, Katherine Lajkosz, Stanley K. Liu, Danny Vesprini, Christopher G. Sherman, Michelle R. Downes

**Affiliations:** 1 Division of Anatomic Pathology, Laboratory Medicine and Molecular Diagnostics, Sunnybrook Health Sciences Centre, Toronto, ON M4N 3M5, Canada; 2 Laboratory Medicine and Pathobiology, University of Toronto, Toronto, ON M5S 1A8, Canada; 3 Department of Biostatistics, Princess Margaret Hospital, Toronto, ON M5G 2C1, Canada; 4 Radiation Oncology, Sunnybrook Health Sciences Centre, Toronto, ON M4N 3M5, Canada

**Keywords:** muscle-invasive bladder cancer, molecular classification, molecular taxonomy, luminal subtype, basal subtype, neuronal subtype, NanoString, gene expression

## Abstract

**Simple Summary:**

Molecular subtyping of muscle-invasive bladder cancer (MIBC) via gene expression can improve therapeutic decision-making and disease prognosis. However, the currently used molecular classification tools are based on complex transcriptomic profiling methodology that hinders timely translation to clinical practice. In this study, we evaluated the NanoString nCounter platform and conventional GATA3-CK5/6 immunohistochemistry for the molecular classification of MIBC in primary care settings. The methodologies were highly concordant and allowed us to explore differences in clinicopathologic parameters and prognosis between intrinsic MIBC molecular subtypes in a cohort of 138 MIBCs.

**Abstract:**

Transcriptional profiling of muscle-invasive bladder cancer (MIBC) using RNA sequencing (RNA-seq) technology has demonstrated the existence of intrinsic basal and luminal molecular subtypes that vary in their prognosis and response to therapy. However, routine use of RNA-seq in a clinical setting is restricted by cost and technical difficulties. Herein, we provide a single-sample NanoString-based seven-gene (KRT5, KRT6C, SERPINB13, UPK1A, UPK2, UPK3A and KRT20) MIBC molecular classifier that assigns a luminal and basal molecular subtype. The classifier was developed in a series of 138 chemotherapy naïve MIBCs split into training (70%) and testing (30%) datasets. Further, we validated the previously published CK5/6 and GATA3 immunohistochemical classifier which showed high concordance of 96.9% with the NanoString-based gene expression classifier. Immunohistochemistry-based molecular subtypes significantly correlated with recurrence-free survival (RFS) and disease-specific survival (DSS) in univariable (*p*  =  0.006 and *p*  =  0.011, respectively) and multivariate cox regression analysis for DSS (*p* = 0.032). Used sequentially, the immunohistochemical- and NanoString-based classifiers provide faster turnaround time, lower cost per sample and simpler data analysis for ease of clinical implementation in routine diagnostics.

## 1. Introduction

Urothelial carcinomas of the bladder have been traditionally classified as either non-muscle-invasive (≤pT1) or muscle-invasive (MIBC, ≥ pT2) [1]. About 75% of newly diagnosed bladder cancers are noninvasive (NMIBC), while the rest are MIBC and are associated with poor outcome, despite aggressive local and systemic treatment [2]. Cystectomy with lymph-node dissection and chemotherapy in the neoadjuvant or adjuvant setting is the standard treatment for MIBC patients [3,4]. Further treatment options such as immune checkpoint inhibitors are now being implemented in a subset of patients with varying success [5]. However, current tools have limited capacity to identify, at time of cystectomy, those that are most at risk of recurrence, metastasis and death of disease. Therefore, identifying which specific subset of MIBC patients would derive the most benefit from these therapies is urgently needed [6,7,8,9,10,11,12,13,14,15,16].

Over the last decade, advances in sequencing technologies have enhanced our understanding of the genomic, transcriptomic and proteomic landscape of MIBC. In particular, RNA sequencing (RNA-seq) revealed that MIBCs can be classified into distinct molecular subtypes with variable clinical properties [17,18,19,20,21]. Multiple different research teams have published various iterations of MIBC molecular subtyping and a consensus definition was recently published tin an attempt to unify these multiple definitions [20,21]. Overall, there is an agreement on at least two general MIBC molecular subtypes, luminal and basal. Importantly, several reports have highlighted the clinical significance of MIBC molecular subtypes, by noting improved survival with neoadjuvant chemotherapy in the basal subtype [17,18] along with enhanced response to immune checkpoint inhibitors in certain luminal subtypes [20,21].

However, these prior efforts have not seen widespread implementation in clinical practice due to their reliance on multi-omic technologies for sample classification, which is neither practical nor fiscally possible in everyday practice. A rapid, cost effective, clinically useful and accurate method of molecular subtyping is therefore needed. Accordingly, immunohistochemical (IHC) markers have been investigated as a clinically applicable alternative to gene expression profiling for MIBC molecular subtyping [22,23,24,25]. We and others have recently reported that CK5/6 and GATA3 IHC can classify MIBCs into basal and luminal molecular subtypes correctly in 80–97% of cases [25].

NanoString nCounter technology provides a lower cost alternative to RNA-seq for gene expression profiling with faster turnaround time and user-friendly readouts for better clinical implementation [26]. Further, the NanoString platform does not require high-quality RNA and thus allows for precise and accurate measurements of RNA expression in formalin-fixed, paraffin-embedded (FFPE) tissue [27]. Therefore, a NanoString-based MIBC molecular classifier is a viable alternative to RNA-seq. Indeed, the utility of a NanoString-based molecular classifier in bladder cancer has been demonstrated in recent publications [28,29].

In this study, we developed a NanoString-based gene expression classifier for molecular stratification of MIBC tumors and assessed its applicability in a clinical setting. Further, we validated a two-marker IHC-based classifier which can be used in routine pathology practice for molecular subtyping of MIBC in primary care centers, building on our previous publication [25].

## 2. Materials and Methods

### 2.1. Case Selection and Morphologic Review

This retrospective study was approved by the Sunnybrook Health Sciences Centre research ethics board (REB 187-2016) and written informed consent was waived. The study included a subset of 138 randomly selected cases from a larger cohort of 243 chemotherapy naïve and checkpoint inhibitor therapy naïve high grade MIBC treated by cystectomy from 1999 to 2019, characterized in our previous study [25]. Cystectomy cases that were < pT2 with non-urothelial histology were excluded. For each case, hematoxylin and eosin slides were reviewed by a pathologist with subspecialty training in genitourinary pathology who confirmed and documented the following: tumor grade (as per 2016 World Health Organization/International Society of Urologic Pathology), pathologic stage (TNM 8th edition), tumor histology, presence of carcinoma in situ (CIS), margin status, lymphovascular invasion and presence of nodal metastases. Date of surgery, neoadjuvant therapy, date of last known follow up, date of recurrence and date of disease-specific death (DSD), if applicable, were recorded. Only deaths noted in the electronic patient record were available.

### 2.2. Tissue Microarray Construction

To perform immunohistochemical staining, tissue microarrays (TMAs) were used as previously described [25]. In brief, for each patient, three tissue sites (superficial, mid and deep tumor) containing MIBC were circled on a single representative slide. Triplicate 1 mm core TMAs were constructed using a TMA instrument (Beecher Instruments, Silver Springs, MD, USA) to punch the areas of interest from the respective FFPE tumor blocks. TMAs were then cut into 4 mm-thick sections for IHC staining.

### 2.3. Immunohistochemistry and Interpretation

GATA3 and CK5/6 expression was evaluated by IHC on TMA sections as previously described [25]. Each case was ascribed as basal (CK 5/6+, GATA3–), luminal (CK 5/6–, GATA3+) or double-negative (CK 5/6–, GATA3–). For cases exhibiting expression of both GATA3 and CK5/6, when CK5/6 exhibited patchy or central loss they were categorized as luminal but when the CK5/6 staining was intense and diffuse, they were classified as basal. Luminal tumors have been reported to occasionally show a linear layer of CK5/6 positive cells outlining the tumor nests and may also have scattered positive CK5/6 cells [22]. Cases were assessed as positive for CK 5/6 and GATA3 when cytoplasmic and nuclear staining were observed, respectively.

### 2.4. RNA Extraction and mRNA Expression Analysis

Tissue RNA extraction from macrodissected FFPE sections (3 to 5 sections per case, 5 µm thickness) was performed using the High-Pure FFPET RNA Isolation Kit (Roche, Basel, Switzerland), following the manufacturer’s instructions. RNA was quantified with QuantusTM Fluorometer (Promega Inc., Madison, WI, USA). RNA profiling was performed with 250 ng of RNA using a custom NanoString probeset (NanoString Technologies Inc, Seattle, WA, USA), following the manufacturer’s instructions. A comprehensive literature review including public databases and published manuscripts was carried out to define a set of differentially expressed genes across MIBC molecular subtypes [9,10,11,12,14,15,16,20,30,31,32]. A custom NanoString nCounter (NanoString Technologies, Seattle, WA, USA) mRNA probe set was created to analyze the expression of 62 mRNA molecules of interest, and also five housekeeping genes. Experimental reagents were provided by NanoString Technologies. Elements TagSet chemistry was utilized. Sequence-specific oligonucleotides with unique tag-attachment sites were designed by NanoString and ordered from an oligonucleotide provider (Integrated DNA Technologies, Inc., Coralville, IA, USA).

Raw reporter code counts and the reporter library file obtained from the nCounter software were utilized for gene expression data analysis using NanoString’s software nSolver version 4.0 with the Advanced Analysis version 2.0 plugin (free software available from NanoString Technologies, Seattle, WA, USA). Data normalization was achieved using internal negative controls, synthetic positive controls and five housekeeping genes. Normalization was performed using geometric mean and data was log2-transformed. Normalized mRNA counts were analyzed for differential expression. Statistically significant, differentially expressed genes (DEGs) were defined as those with expression levels corresponding to a fold change > 2 and Benjamini–Hochberg (BH) adjusted *p* < 0.05 (to control the false discovery rate (FDR)). Unsupervised hierarchical clustering and heatmap visualization was performed using the using the R package ComplexHeatmap (version 2021.9.2.382). Subtype classification was run using a PAM classifier with the pamr package version 1.56.1 in R Studio version 4.0.0 to derive both molecular subtype calls and correlations to the basal and luminal centroid for each case. The data were split into training and test datasets using a 70/30 split ratio, with five-fold cross-validation used to select the optimal threshold in the training dataset estimate expected performance.

### 2.5. Statistical Analysis

Statistical analysis was performed using SPSS 24.0 (IBM Corporation, Armonk, NY, USA). Data correlations were assessed using Chi-Square test for categorical variables and one-way ANOVA for continuous variables. Survival curves were calculated by the log-rank test and visualized with the Kaplan–Meier plot for recurrence free survival (RFS) and disease specific survival (DSS). Univariate and multivariate Cox proportional hazards regression model was used to compute the prognostic value of the molecular classifiers with regard to RFS and DSS. A two-sided *p* < 0.05 was deemed statistically significant.

## 3. Results

### 3.1. Development of NanoString nCounter Probe Set for Muscle-Invasive Bladder Cancer Molecular Stratification

To define MIBC molecular groups, we performed NanoString-based gene expression profiling of FFPE tissue from a cohort of 138 chemotherapy naïve MIBC (≥ pT2) patients, divided into Cohort I (*n* = 72) and Cohort II (*n* = 66). Samples were selected on the basis of availability of tissue as well as quantity of RNA for analyses. Clinicopathological parameters of the cohort are summarized in Supplementary Table S1.

In the development phase of the study, we custom designed a NanoString nCounter probe set comprised of 62 literature-curated candidate genes with the aim of developing mRNA signatures specific for the luminal, basal and neuroendocrine MIBC molecular subtypes. Neuroendocrine markers have been previously suggested to define a group of MIBCs that are negative for basal and luminal markers and, consequently, was included in this study [32]. The 62 candidate genes were selected based on a comprehensive literature review, including MIBC-associated genes and MIBC subtyping models described by the University of North Carolina (UNC), MD Anderson Cancer Center (MDA), The Cancer Genome Atlas (TCGA), Lund and Baylor [9,10,11,12,14,15,16,20,30,31,32] (Supplementary Table S2). We employed this 62-gene set to profile Cohort I (*n* = 72).

**Figure 1:**
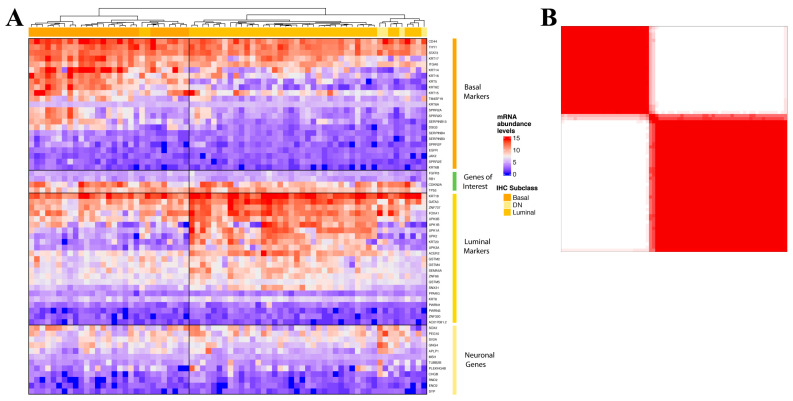
(**A**) Unsupervised hierarchical clustering (Pearson correlation) of normalized abundance levels of 62 mRNAs derived by NanoString nCounter assay for Cohort I. Annotation of immunohistochemical subtypes is indicated for reference; (**B**) consensus matrix (k  =  2) for Cohort I where samples represent rows and columns. The consensus values range from 0 denoted in white (never clustered together) to 1 denoted in red (always clustered together).

Unsupervised hierarchical clustering of gene expression levels using Pearson correlation revealed two different gene clusters, each defined by distinct patterns of expression of luminal and basal markers (Figure 1A). The first cluster, referred to as basal (*n* = 30), expressed higher levels of basal cytokeratins and other genes associated with urothelial basal cells (such as DSG3, KRT14 and CD44), while expression of urothelial cell differentiation genes was relatively lower. The second cluster, referred to as luminal (*n*  =  42), expressed relatively lower levels of basal genes and higher levels of terminal urothelial differentiation genes such as KRT20, GATA3 and FOXA1. Further, consensus clustering analysis also yielded the same two clusters, suggesting that two robust expression subtypes, luminal and basal, exist in our cohort (Figure 1B). Differential gene expression analysis utilizing linear regression analysis with multiple-testing correction identified 18 DEGs between basal and luminal tumors according to the cutoff values of |Fold Change| ≥ 2 and FDR *p*  ≤  0.05 (Supplementary Table S3).

### 3.2. Validation of the Muscle-Invasive Bladder Cancer NanoString-Based Gene Expression Classifier

**Figure 2:**
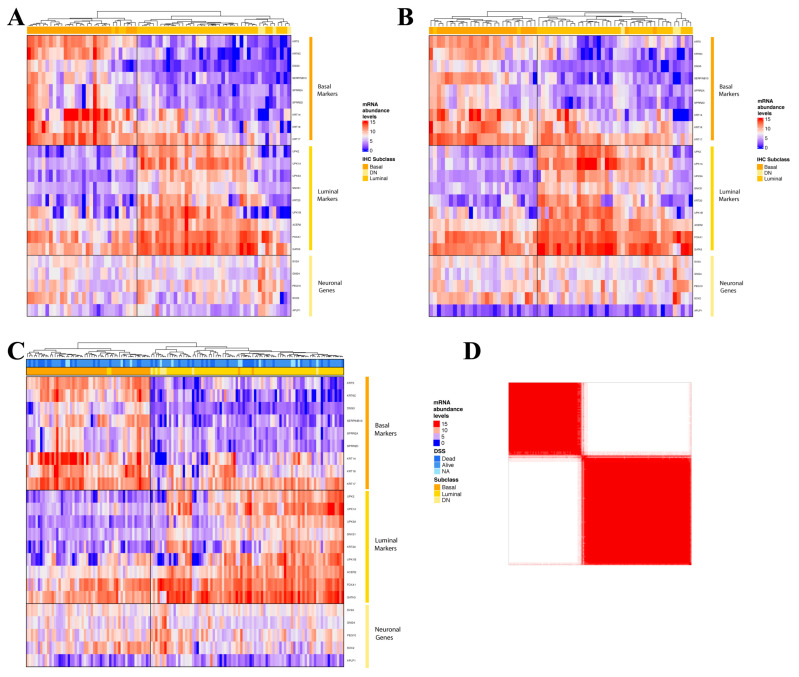
Unsupervised hierarchical clustering (Pearson correlation) of normalized abundance levels of a filtered list of 23 mRNAs derived by NanoString nCounter assay for (**A**) Cohort I; (**B**) Cohort II; and (**C**) total cohort of 138 tumors. Annotation of immunohistochemical subtypes and disease specific survival is indicated for reference. (**D**) Consensus matrix (k  =  2) for the total cohort of 138 tumors. Samples represent both rows and columns, and consensus values range from 0 denoted in white (never clustered together) to 1 denoted in red (always clustered together).

In the validation phase of the study, we custom designed a NanoString nCounter probe set comprised of a filtered list of genes including the 18 DEG from the development phase and five neuronal genes (to help define potential neuroendocrine MIBCs, Supplementary Table S2). Unsupervised hierarchical clustering of these select genes expression levels in Cohort I using Pearson correlation yielded the same basal and luminal clusters we identified in the development phase (Figure 2A). This 23-gene panel was then used to profile Cohort II (n = 66). Utilizing unsupervised hierarchical clustering we confirmed the presence of luminal and basal subtypes of MIBC (Figure 2B). Taken together, unsupervised and consensus clustering of the 23 markers showed that our cohort of 138 tumors can be divided into two clusters; luminal (*n* = 84) and basal (*n* = 54) that show an opposing transcriptomic profile (Figure 2C,D).

### 3.3. Immunohistochemical Muscle-Invasive Bladder Cancer Molecular Classifier

To define IHC-based MIBC molecular groups, we investigated the previously published routine assays for GATA3 and CK5/6 [22]. Both GATA3 and CK5/6 showed a strong positive correlation between mRNA expression and the respective encoded protein expression status (GATA3 AUC = 0.887 and CK5/6 AUC = 0.961).

Assessment of the IHC slides with the knowledge of NanoString-based molecular subtypes provided the following insights into the identification of IHC-based MIBC molecular subtypes; strong uniform positivity (++) for CK 5/6 was always indicative of basal tumors, irrespective of GATA3 staining patterns. In cases where CK 5/6 expression was less than ++, including either central loss of staining or complete loss of staining, then any GATA3 staining pattern was indicative of a luminal tumor. This IHC-based classification algorithm was then provided as a written instruction without any case review to a second genitourinary pathologist (CS) who independently reviewed 60 cases in a blinded fashion. There was agreement in 57/60 cases (95%).

**Figure 3:**
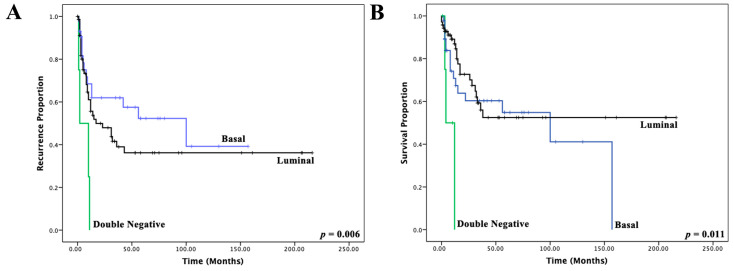
(**A**) Kaplan–Meier plots and log-rank *p*-values correlating the three immunohistochemical molecular subtypes with recurrence-free survival; (**B**) and disease-specific survival.

The final IHC-based classification was as follows: basal n = 54, luminal n = 77 and double-negative n = 7. Assignments to these molecular subtypes significantly correlated with RFS and DSS in univariable Kaplan–Meier regression (Figure 3A,B, log-rank *p*  =  0.006 and *p*  =  0.011, respectively) and multivariate cox regression analysis for DSS (*p* = 0.032, Table 1).

Comparing the IHC classifier to the NanoString 23-gene expression clustering, a high level of agreement was observed (Kappa = 0.937), with only four (3.1%) cases assigned a different molecular subtype by the IHC classifier versus gene expression clustering. Of note, cases staining negative for both CK5/6 and GATA3 on IHC and were classified as double-negative did not form a distinct cluster based on their gene expression but rather clustered with luminal MIBCs. Therefore, double-negative cases were excluded from comparative analysis between IHC and NanoString 23-gene expression clustering. 

### 3.4. Development of a NanoString-Based MIBC Molecular Classifier

**Figure 4:**
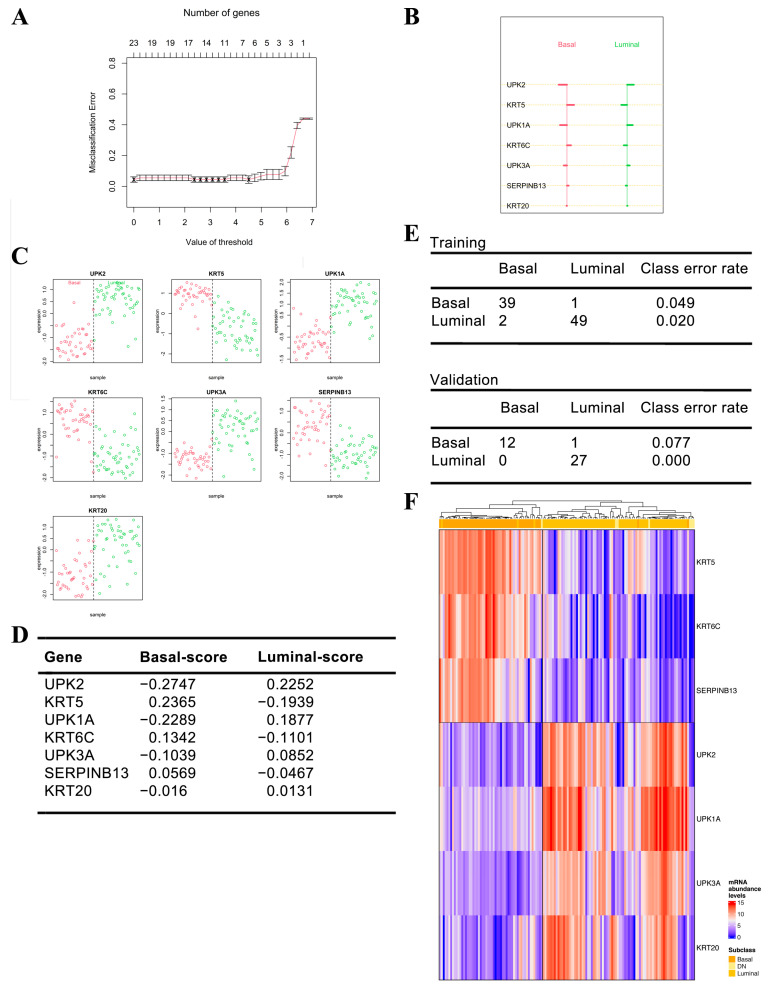
Nearest shrunken centroids classification. (**A**) Illustration of training the PAMR prediction profile within the training dataset. At threshold 4.51, the misclassification error rate was minimal, resulting in seven genes being selected for the classifier (KRT5, UPK2, KRT6C, UPK1A, SERPINB13, UPK3A, KRT20); (**B**) visualization of the shrunken class centroids, i.e., the distance of each gene to the nearest shrunken centroid for each subtype; (**C**) dot plot of the relationship between gene expression and MIBC subtype classification for each tumor in the training dataset. Each point represents a unique tumor and the color represents the MIBC subtype that the PAM classifier classified these tumors into; (**D**) PAMR scores for each gene in the seven-gene MIBC molecular classifier; (**E**) confusion matrix for predicted subtype vs. immunohistochemical subtype. The overall misclassification error rate was 3.3% in the training dataset and 2.5% in the testing dataset; (**F**) unsupervised hierarchical clustering (Pearson correlation) of normalized abundance levels of the seven genes selected for the PAM classifier in the total cohort of 138 tumors.

To train an accurate and robust NanoString-based MIBC molecular classifier we utilized PAM (prediction analysis of microarrays), a statistical approach to class prediction from gene expression data via nearest shrunken centroid method. First, we utilized PAM to identify the number of genes that best characterize the basal and luminal centroids using the IHC classifier as the supervising variable. For this, the combined cohort was randomly split into training (*n* = 91) and test (*n* = 40) datasets using a 70/30 split ratio. Within the training dataset, we established a seven-gene PAM classifier by setting the optimal threshold for centroid shrinkage taking into account the trade-off between classification performance and gene signature size (Figure 4A). The following genes were selected as surrogate markers for the basal (KRT5, KRT6C and SERPINB13) and luminal (UPK1A, UPK2, UPK3A and KRT20) MIBC subtypes (Figure 4B–E). Application of the classifier to the training dataset was highly accurate as only 3 of the 91 samples (3.3%) were discordant between the IHC and NanoString-based gene expression classifier (Figure 4D). When applied to the test dataset, only 1 of the 40 samples (2.5%) was discordant between the IHC and NanoString-based MIBC molecular classifier (Figure 4F).

Next, we examined the clinical characteristics of the two molecular subtypes (Table 2) and found association of CIS and LVI with luminal subtype classification (*p* < 0.05).

**Figure 5:**
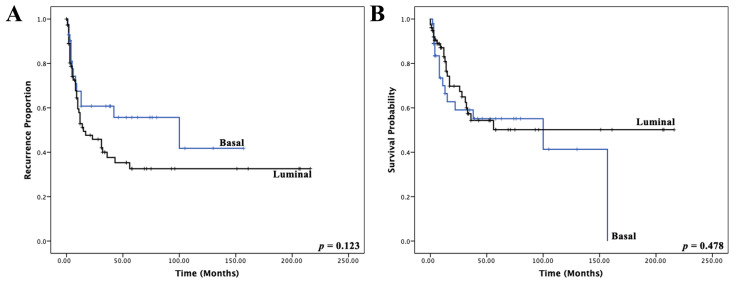
(**A**) Kaplan–Meier plots and log-rank *p*-values correlating the two NanoString-based gene expression molecular subtypes with recurrence-free survival (**B**) and disease-specific survival.

Lastly, we explored the correlation between NanoString-based MIBC molecular subtypes and patient survival. Figure 5 illustrates a univariate Kaplan–Meier regression for RFS and DSS and Table 1 the univariate and multivariate Cox proportional hazards model with DSS as the end point. Although patients with a basal MIBC had a shorter median time to recurrence (8.5 months versus 10.0 months for luminal MIBCs) and DSD (4.5 months versus 12 months for luminal MIBCs), it did not reach statistical significance (*p* > 0.05).

### 3.5. Technical Cost Analysis

The reagent cost of one NanoString assay testing 23 mRNAs and five housekeeping genes was 87.53 CAD per sample. The NanoString-based gene expression classifier assay with seven mRNAs and five housekeeping genes is estimated to cost 60.99 CAD. Lab fee was 30.42 CAD per sample. Cutting unstained sections was 10.20 CAD and RNA extraction was 20 CAD per sample. Therefore, the total per sample cost for the NanoString assay was 148.14 CAD while the cost of a two-antibody IHC panel (CK 5/6 and GATA3) was 29.24 CAD per sample.

## 4. Discussion

The introduction of molecular subtype classification has advanced our understanding of MIBC and has shown great potential to improve the diagnostics and treatment of MIBC in the future. However, it is based on whole transcriptome analysis which is challenging to implement in routine clinical diagnostics. Therefore, surrogate markers of MIBC molecular subtypes are needed.

In this study, we have developed and validated a MIBC molecular classifier that accurately stratifies FFPE samples using a seven-gene expression panel quantified by the NanoString nCounter platform. Further, we validated the previously published GATA3 and CK5/6 IHC classifier which showed high concordance (96.9%) with gene expression-based MIBC molecular subtyping. These results validate the utility of IHC for MIBC molecular subtyping [22,33]. In light of our present findings and previous reports we suggest implementing this clinically applicable two-marker IHC panel in routine diagnostics to identify the intrinsic basal, luminal and double-negative molecular subtypes of MIBC [22,33]. Cases that may be difficult to classify such as the ones displaying unusual patterns of GATA3 and/or CK5/6 expression or cases with ambiguous staining pattern would then be sent for confirmatory gene expression-based testing. Future studies with larger cohorts are necessary to explore if discordant classifications between gene expression-based and immunohistochemistry-based subtyping are clinically relevant.

Current MIBC molecular classification schemas, including the recently published consensus molecular classification, are based on whole transcriptome profiling [20]. This hinders the translation of MIBC molecular subtype classification into routine clinical practice as the technology is expensive, time consuming, requires high quality RNA and extensive bioinformatics resources. To surmount these limitations as well as the limited availability of RNA sequencing technology in primary care settings, we conducted gene expression analysis using the NanoString nCounter platform. The NanoString assay provides a faster turnaround time and lower cost per sample while maintaining the accuracy of whole transcriptome-based subtype classifiers [28,29]. Kardos et al. performed comparative analysis of the RNA-Seq and NanoString platforms for a two subtype (basal and luminal) molecular stratification of MIBC utilizing a 47-gene classifier, BASE47 [29]. The NanoString-based BASE47 MIBC molecular classifier had an accuracy of 87% and 93% in the training and validation datasets, respectively. Another study by Weyerer et al. utilized a NanoString-based modified 21-gene MDA MIBC molecular classifier and a 6 marker IHC classifier to analyze a cohort of 193 MIBCs [23]. The study defined four distinct cluster groups; basal, luminal, luminal p53-/ECM-like and double-negative. There was 83.9% concordance between gene expression-based and IHC-based subtyping. Additionally, Lopez-Beltran et al. reported a four-gene NanoString-based classifier for a three-subtype molecular stratification; basal, luminal and null/double-negative [28]. This classifier was utilized for molecular stratification of 91 bladder urothelial carcinomas (including NMIBCs and MIBCs), which had prognostic implications. In line with these studies, our findings further support the utility of the NanoString nCounter platform as an accurate tool for MIBC molecular classification. Notably, there was an overlap between the genes included in our seven-gene NanoString-based panel and each of the abovementioned previously published panels including the established basal markers KRT5 and KRT6C as well as the luminal markers UPK2 and KRT20.

Our NanoString-based gene expression MIBC classifier has several advantages for easier clinical implementation over whole transcriptome clustering analysis. First, it can stratify a single individual sample. The PAM classifier developed in this study does not compare relative gene expression patterns among tumors, thus eliminating the need for platform normalization and/or a cohort of samples for subtyping algorithm development. Second, NanoString technology provides a more affordable, fast, and less complex, more clinically accessible results. Moreover, MIBC subtype classification based on IHC presents similar advantages to the NanoString platform and thus has gained interest in the field. Guo et al. has reported that GATA3-CK5/6 IHC-based MIBC classifier has > 80% accuracy [22]. Other studies since used these markers for assessment of molecular subtypes of MIBC and found them to be an accurate tool for MIBC tumor classification that could be eventually implemented in a clinical setting [23,28,33,34,35]. Our findings are consistent with these reports. In our cohort of 138 MIBCs, we found a 96.9% concordance between gene expression-based and protein-based subtyping. The importance of this is that most MIBC should be readily classified as basal or luminal using IHC with only difficult to classify cases requiring a NanoString approach. One additional advantage to IHC is the ability to further subclassify the heterogeneous luminal group using p16 IHC into genomically unstable (GU) and urothelial-like (Uro-like) subtypes which adds prognostic information [25].

Guo et al. and Koll et al. have previously reported a molecular subtype of MIBC characterized by lack of basal or luminal IHC marker expression [22,33]. Similarly, a small fraction of tumors in our cohort did not express either basal or luminal IHC markers and thus were referred to as double-negative. This molecular subtype seems to be of clinical significance, as patients with double-negative MIBC in our cohort had decreased DSS rates in univariate and multivariate analysis, which is in concordance with previously published data [23,24,33]. Weyerer et al. recently reported an association between the IHC double-negative MIBC subtype and neuroendocrine tumor histology [23]. Further, in a study by Koll et al., all cases with neuroendocrine histology were double-negative for CK5/6 and GATA3 IHC [33]. Neuroendocrine bladder carcinoma is a rare variant of MIBC associated with poor prognosis [32]. This could potentially explain the association between the IHC double-negative MIBC subtype and inferior outcomes. To identify neuroendocrine tumors in our cohort we have added various neuronal markers (i.e., NESTIN, TUBB2B, PEG10) to our gene classifier. However, MIBCs double-negative for CK5/6 and GATA3 IHC in our cohort did not form a cluster with a distinct gene expression profile characterized by high neuronal gene expression combined with low basal and luminal gene expression as previously reported [32]. In our cohort, IHC double-negative MIBCs clustered with luminal MIBCs in gene expression analysis and were classified as such using the NanoString-based gene expression classifier. This highlights the heterogeneity of IHC double-negative MIBCs.

The potential of MIBC molecular subtyping to improve precision patient management and survival outcomes has been documented in the literature [17,20]. Therefore, the potential benefit of MIBC molecular stratification is that the subtype may represent an informative description of tumor biology that translates into improved risk stratification and clinical decision-making compared to grade and stage alone. However, contradictory results with regard to outcome have been published, partially attributed to the diversity of molecular subtype taxonomy in MIBC [20]. In the recently published consensus molecular classifier, Kamouns et al. analyzed the transcriptome of 1750 MIBCs and found significant prognostic differences only between the luminal and neuroendocrine-like MIBC molecular subtype, known to have aggressive clinical behavior [20]. Further, Kollberg et al. did not find molecular subtyping to be a prognostic factor in a population-based consecutive cystectomy cohort of 519 patients [36]. Our findings are in line with these previous studies as we demonstrate no association between gene expression-based molecular subtyping and DSS or RFS. In our study, only IHC double-negative MIBCs had significantly worse prognosis compared to luminal MIBCs. The lack of difference in prognosis between basal and luminal molecular subtypes in the present study could be attributed to variation in prognosis among the different previously published sub-classes within the luminal and basal classifications [37] as we have previously shown that IHC subclassification of the luminal group into GU and Uro-like is prognostically significant. Future prospective multicenter trials are needed to investigate the prognostic impact of higher-resolution MIBC subtyping in cohorts with clinically meaningful outcomes.

This retrospective study has several limitations. First, the sample size in this study was relatively small and thus may be not sufficiently powered to demonstrate association with survival, as has been the case with prior classifiers in MIBC. Second, lack of sufficient number of patients with neoadjuvant treatment in our cohort hindered our ability to analyze the possible predictive value of molecular subtyping in MIBC. Therefore, our classifier will require additional prospective validation before it can be used clinically. Nonetheless, given the low classification error in our training and testing datasets, it advances molecular subtype classification toward clinical utility.

## 5. Conclusions

In conclusion, the development of a simplified two-marker immunohistochemical MIBC molecular classifier to be used sequentially with a seven-gene NanoString assay provides an accurate and clinically applicable approach to MIBC molecular subtyping. Further, this approach may accelerate future research in the field and the eventual implementation of MIBC molecular subtype classification into routine clinical use.

## Figures and Tables

**Table 1:** Univariate and multivariate analysis of clinic-pathological parameters related to cancer-specific survival prediction in the current study.

	Univariate		Multivariate	
Variable	HR (95% CI)	*p*-Value	HR (95% CI)	*p*-Value
Age ≥ 74 (median)	1.179 (0.637–2.183)	0.600		
Stage pT4	2.111 (1.104–4.038)	0.024	1.204 (0.548–2.644)	0.644
Positive surgical Margins	2.228 (1.152–4.309)	0.017	1.985 (0.881–4.474)	0.098
Lymph Node Involvement	2.335 (1.232–4.425)	0.009	2.251 (1.132–4.476)	0.021
Variant histologic subtype	1.516 (0.781–2.945)	0.219		
NanoString-based gene expression subtype				
Basal vs. Luminal	1.125 (0.670–2.336)	0.482		
Immunohistochemical subtype				0.032
Double-Negative vs. Luminal	5.868 (1.607–21.427)	0.007	5.326 (1.372–20.669)	0.016
Basal vs. luminal	1.734 (0.864–3.480)	0.122	1.714 (0.853–3.443)	0.130

HR, hazard ratio; 95% CI, 95% confidence interval.

**Table 2:** Relationship between molecular subtypes and clinicopathological parameters of 138 muscle-invasive bladder cancers included in the study.

Variables	Study Cohort			
	Total	Basal	Luminal	χ2
	*n* = 138	*n* = 52	*n* = 86	*p*-Value
Sex				0.939
Female	35 (25%)	13 (37%)	22 (63%)	
Male	103 (75%)	39 (38%)	64 (62%)	
Age	72.1 (33–90)	73.4 (49–90)	71.4 (33–88)	0.277
Histology				0.144
Urothelial carcinoma	99 (72%)	37 (37%)	62 (63%)	
Squamous	23 (17%)	13 (57%)	10 (43%)	
Sarcomatoid	4 (3%)	1 (25%)	3 (75%)	
Nested	3 (2%)	0	3 (100%)	
Micropapillary	4 (3%)	0	4 (100%)	
Plasmacytoid	4 (3%)	1 (25%)	3 (75%)	
Carcinoma in situ				0.003
Present	56 (41%)	13 (23%)	43 (77%)	
Absent	81 (59%)	39 (48%)	42 (52%)	
Stage				0.859
pT2	15 (11%)	5 (33%)	10 (67%)	
pT3	81 (59%)	32 (40%)	49 (60%)	
pT4	42 (30%)	15 (36%)	27 (64%)	
Node				0.138
N0	88 (64%)	37 (42%)	51 (58%)	
N1	45 (33%)	13 (29%)	32 (71%)	
N/A	5 (4%)	2 (40%)	3 (60%)	
Margins				0.377
No	103 (75%)	41 (40%)	62 (60%)	
Yes	35 (25%)	11 (31%)	24 (69%)	
Lymphovascular invasion				0.033
No	41 (30%)	21 (51%)	20 (49%)	
Yes	97 (70%)	31 (32%)	66 (68%)	
Recurrence				0.058
No	62 (45%)	27 (44%)	35 (56%)	
Yes	56 (41%)	15 (27%)	41 (73%)	
N/A	20 (14%)	10 (50%)	10 (50%)	
Death				0.623
No	84 (61%)	31 (37%)	53 (61%)	
Yes	41 (30%)	17 (41%)	24 (59%)	
N/A	13 (9%)	4 (31%)	9 (69%)	

Note: The patients in this cohort did not receive any chemotherapy or checkpoint inhibitor therapy prior to their cystectomy. Postoperative chemotherapy was given in 32 patients and post-operative immune checkpoint therapy in 3 patients.

## Data Availability

Data available on request due to privacy restrictions.

## Supplementary Materials

The following supporting information can be downloaded at: https://www.mdpi.com/article/10.3390/cancers14194911/s1, Table S1: Clinicopathologic characteristics of Cohorts I and II; Table S2: Genes used in NanoString-based gene expression profiling (*n* = 62); Table S3: List of 18 differentially expressed genes between basal and luminal tumors according to NanoString-based gene expression hierarchical clustering and 5 neuronal genes used in the study to help define potential neuroendocrine muscle-invasive bladder cancers.
