# Supplementary material for: Development of a Clinically Applicable NanoString-Based Gene Expression Classifier for Muscle-Invasive Bladder Cancer Molecular Stratification
Source: Cancers (Basel). 2022 Oct 7;14(19):4911. doi: 10.3390/cancers14194911 (PMC9564169; doi:10.3390/cancers14194911)
Supplement: Supplementary file 1 [file cancers-14-04911-s001.zip › cancers-1903758-supplementary.pdf]

Supplementary Tables

Table S1. Clinicopathologic characteristics of Cohorts I and II.

	**Total (*N* = 138)**	**Cohort I (*n*=72)**	**Cohort II (*n*=66)**	***p* -Value**
Age (years)				0.098
Mean (Range)	72.1 (33-90)	70.6 (33-88)	73.6 (49-90)	
**Sex**				0.585
Male	103 (75)	52 (73)	51 (77)	
Female	34 (25)	19 (27)	15 (23)	
CIS				0.994
Present	56 (41)	29 (41)	27 (41)	
Absent	81 (59)	42 (59)	39 (59)	
pT Category				0.742
pT2	14 (10)	6 (9)	8 (12)	
pT3	81 (59)	42 (59)	39 (59)	
pT4	42 (31)	23 (32)	19 (29)	
**Margins**				0.466
Negative	102 (74)	51 (71)	51 (77)	
Positive	35 (25)	20 (28)	15 (23)	
N/A	1 (1)	1 (1)		
Lymphovascular invasion				0.393
Absent	40 (29)	23 (32)	17 (26)	
Present	97 (70)	48 (67)	49 (74)	
N/A	1 (1)	1 (1)		
Lymph Nodes				0.161
Negative	40 (63)	41 (57)	46 (70)	
Positive	98 (33)	27 (38)	18 (27)	
N/A	6 (4)	4 (6)	2 (3)	
Event-free survival				0.046
Relapse	56 (41)	39 (54)	17 (24)	
No Relapse	62 (45)	32 (44)	30 (42)	
N/A	20 (14)	1 (1)	19 (26)	
Death	41 (30)	25 (35)	16 (24)	0.510
No Death	84 (61)	46 (64)	38 (58)	
N/A	13 (9)	1 (1)	12 (18)	
Follow-up time (months)				
Relapse				
Mean (Range)	30.2 (0-216)	30.6 (0-206)	29.5 (0-216)	0.902
Death				
Mean (Range)	27.6 (0-216)	32.2 (0-206)	22.5 (0-216)	0.183

CIS, Carcinoma In Situ; pT, Pathologic T category; N/A, not available.

Note: The patients in this study did not receive any chemotherapy or checkpoint inhibitor therapy prior to their cystectomy. Postoperative chemotherapy was given in 32 patients and postoperative immune checkpoint therapy in 3 patients.

Table S2. Genes used in NanoString-based gene expression profiling (*n*=62).

Gene name	Reference
THY1	[15], [20]
STAT3	[15], [38]*
JAK2	[15]
KRT15	[15], [39]*
EGFR	[11], [15], [16]
ITGA6	[15]
KRT14	[10], [12], [14], [15], [16]
KRT5	[10], [11], [12], [14], [15], [16]
KRT17	[9], [15]
CD44	[10], [11], [12], [14], [15]
UPK1B	[15], [16]
UPK1A	[14], [15], [16], [20], [30]
UPK3A	[15], [16]
UPK2	[9], [30], [14], [15], [16], [30]
UPK3B	[15]
KRT20	[10], [12], [14], [15], [16], [20]
KRT18	[10], [12], [15]
KRT8	[10], [15]
PWRN1	[32]
PWRN3	[32]
GSTM5	[32]
GSTM4	[32]
GSTM2	[32]
ZNF320	[32]
FGFR3	[14]
ZNF66	[32]
ZNF737	[32]
FOXA1	[32], [10], [12], [11], [14], [16]
AC017081.2	[32]
ACER2	[32]
SEMA5A	[9], [20], [30], [32]
PPARG	[9], [10], [14], [16], [20], [30], [32]
GATA3	[9], [10], [16], [30], [32]
SNX31	[14], [20], [30], [32]
TM4SF19	[32]
SERPINB13	[20], [32]
SERPINB3	[20], [32]
SERPINB4	[20], [32]
SPRR2F	[32]
SPRR2E	[32]
SPRR2A	[32]
SPRR2D	[32]

KRT16	[10], [20], [32]
DSG3	[20], [32]
KRT6C	[10], [32]
KRT6A	[10], [14], [20], [32]
KRT6B	[10], [11], [20], [30], [32]
PLEKHG4B	[14], [32]
GNG4	[14], [32]
PEG10	[14], [32]
SOX2	[14], [32]
TUBB2B	[14], [32]
CHGB	[32]
SYP	[16], [32]
ENO2	[16], [32]
SV2A	[32]
MSI1	[14], [32]
RND2	[14], [32]
APLP1	[14], [32]
CDKN2A	[16], Internal work
RB1	[16], Internal work
TP53	[12], Internal work

38*. Mo, Q.; Li R.; Adeegbe DO.; Peng G.; Chan KS. Integrative multi-omics analysis of muscle-invasive bladder cancer identifies prognostic biomarkers for frontline chemotherapy and immunotherapy. *Commun Biol* **2020**, *3*,784.

39*. Eich, ML.; Dyrskjøt ,L.; Netto, GJ. Toward personalized management in bladder cancer: the promise of novel molecular taxonomy. *Virchows Arch* **2017**, *471*, 271-280.

Table S3. List of 18 differentially expressed genes between basal and luminal tumors according to NanoString-based gene expression hierarchical clustering and 5 neuronal genes used in the study to help define potential neuroendocrine muscle invasive bladder cancers.

Gene	FDR(BH)	Fold Change	Upregulated In
KRT5	0.001	5.6	Basal
KRT6C	0.001	5.2	Basal
DSG3	0.001	3.5	Basal
KRT16	0.001	2.9	Basal
KRT14	0.002	4.4	Basal
KRT17	0.006	2.1	Basal
SERPINB13	0.007	2.4	Basal
SPRR2A	0.012	2.3	Basal
SPRR2D	0.014	2.3	Basal
UPK2	0.001	4.8	Luminal
UPK1A	0.001	4.4	Luminal
UPK3A	0.001	3.8	Luminal
KRT20	0.001	3.6	Luminal
GATA3	0.001	3.4	Luminal
ACER2	0.001	2.7	Luminal
SNX31	0.001	2.1	Luminal
UPK1B	0.012	2.9	Luminal
FOXA1	0.013	2.0	Luminal
SOX2*	0.302	1.1	
GNG4*	0.977	0.1	
APLP1*	0.993	0.0	
SV2A*	0.993	0.0	
PEG10*	0.211	0.9	

* Neuronal genes
